# Fabrication of Waterborne Silicone-Modified Polyurethane Nanofibers for Nonfluorine Elastic Waterproof and Breathable Membranes

**DOI:** 10.3390/polym16111505

**Published:** 2024-05-25

**Authors:** Fang Li, Kai Weng, Toshihisa Tanaka, Jianxin He, Haimin Zheng, Daisuke Noda, Shinji Irifune, Hiromasa Sato

**Affiliations:** 1 Interdisciplinary Graduate School of Science and Technology, Shinshu University, 3-15-1, Tokida, Ueda-shi 386-8567, Nagano, Japan; 20hs155a@shinshu-u.ac.jp (F.L.); 20hs152f@shinshu-u.ac.jp (K.W.); 2 International Joint Laboratory of New Textile Materials and Textiles of Henan Province, Zhongyuan University of Technology, Zhengzhou 450007, China; 3 Silicone-Electronics Materials Research Center, Shin-Etsu Chemical Co., Ltd., 1-10, Hitomi, Matsuida-Machi, Annaka-shi 379-0224, Gunma, Japan; 4 Dainichiseika Color & Chemicals Mfg. Co., Ltd., 2087-4, Ohta, Sakura-shi 285-0808, Chiba, Japan

**Keywords:** waterborne silicone-modified polyurethane, nanofibrous membrane, crosslinking agent, fluorine-free, elasticity, waterproof, breathable

## Abstract

Waterproof and breathable membranes have a huge market demand in areas, such as textiles and medical protection. However, existing fluorinated nanofibrous membranes, while possessing good waterproof and breathable properties, pose health and environmental hazards. Consequently, fabricating fluorine-free, eco-friendly waterborne membranes by integrating outstanding waterproofing, breathability, and robust mechanical performance remains a significant challenge. Herein, we successfully prepared waterborne silicone-modified polyurethane nanofibrous membranes with excellent elasticity, waterproofing, and breathability properties through waterborne electrospinning, using a small quantity of poly(ethylene oxide) as a template polymer and in situ doping of the poly(carbodiimide) crosslinking agent, followed by a simple hot-pressing treatment. The silicone imparted the nanofibrous membrane with high hydrophobicity, and the crosslinking agent enabled its stable porous structure. The hot-pressing treatment (120 °C) further reduced the pore size and improved the water resistance. This environmentally friendly nanofibrous membrane showed a high elongation at break of 428%, an ultra-high elasticity of 67.5% (160 cycles under 400% tensile strain), an air transmission of 13.2 mm s^−1^, a water vapor transmission rate of 5476 g m^−2^ d^−1^, a hydrostatic pressure of 51.5 kPa, and a static water contact angle of 137.9°. The successful fabrication of these environmentally friendly, highly elastic membranes provides an important reference for applications in healthcare, protective textiles, and water purification.

## 1. Introduction

Waterproof and breathable membranes (WBMs) are a class of functional materials with the ability to prevent the penetration of liquid water and allow air and water vapor to pass through and have a wide range of applications and market demand in a variety of fields, such as outdoor apparel, medical protection, oil and water separation, and smart wearables [1,2,3,4,5]. WBMs can be classified as hydrophilic nonporous membranes and hydrophobic porous membranes according to their working principle [6,7,8]. Hydrophilic nonporous membranes show excellent waterproofness due to their non-porous structure. Water vapor molecules are transported through these membranes via a process of ‘adsorption-diffusion-desorption’ facilitated by their hydrophilic groups. However, these membranes do not allow the passage of air, which limits their range of applications. The water repellency of hydrophobic microporous membranes is mainly achieved by the Laplace added pressure generated by their tiny pore structure. Due to the presence of numerous interconnected channels that facilitate the passage of air and the rapid transfer of water vapor molecules, they exhibit excellent water repellency and air permeability and are therefore receiving more and more attention [3,9,10]. Hydrophobic microporous membranes are generally classified into fluorinated membranes and non-fluorinated membranes. Although fluorinated membranes have good water repellency and air permeability, perfluoro octane sulphonates and perfluorooctanoic acid, which are often used in the preparation of fluorinated membranes, have been identified as Persistent Organic Pollutants (POPs). Their incidental toxicity can accumulate in the environment and may have adverse effects on the human body, including potential harm to the immune system, liver, and thyroid. And the harmful gases produced via high-temperature decomposition can cause serious harm to the human respiratory system [8,11]. Therefore, there is an urgent need to develop a fluorine-free elastomeric microporous membrane with green sustainability, waterproof, and breathable properties.

The electrospinning technology is capable of preparing three-dimensional porous nanofibrous membranes with a small diameter, small pore size, and high porosity, and its simple operation and controllable process makes it promising for WBMs [3,10,12,13]. So far, a variety of polymers have been used as raw materials to fabricate nonfluorine nanofibrous membranes exhibiting waterproof and moisture-permeable properties [14,15,16]. Their hydrophobicity is generally obtained through either post-coating treatment or by incorporating low-surface-energy silanes mixed during one-step electrospinning. The former usually involves covering the surface of the nanofibrous membrane with low-surface-energy chemicals through dip-coating to enhance the waterproofness [4,14,15,17,18]. For example, Gu et al. [18] obtained a water resistance of 73.6 kPa and a water vapor transmission (WVT) rate of 9.03 kg m^−2^ d^−1^ by immersing polyurethane/poly(ε-caprolactone) (PU/PCL) nanofibrous membranes in a polydimethylsiloxane (PDMS) coating. Zhao et al. [15] prepared poly(styrene-b-butadiene-b-styrene) (SBS) nanofibers via electrospinning. By dipping SiO_2_ nanoparticles and using polydopamine (PDA) as a binder, the prepared SBS/PDA/SiO_2_ nanofibrous membranes exhibited a hydrostatic pressure of 84.2 kPa and a WVT rate of 6.4 kg m^−2^ d^−1^ (inverted cup method). However, the improvement of water resistance inevitably leads to the blockage of some pores inside the nanofibrous membrane, forming a dense structure and reducing the air permeability. Therefore, researchers have continuously attempted to obtain WBMs by using a one-step electrospinning technique. Gu et al. [19] obtained WBMs with maximum WCA (142 ± 1°), good hydrostatic pressure (5.45 kPa), and WVT rate (8.05 kg m^−2^ d^−1^) by blending a polyurethane/hydrophobic silica gel (PU/HSG). You et al. [20] obtained WBMs with excellent hydrostatic pressure (86.9 kPa) and a good WVT rate (5.1 kg m^−2^ d^−1^) via the one-step electrospinning of polyethylene terephthalate/polydimethylsiloxane (PET/PDMS). Unfortunately, these conventional fibrous membranes are usually fabricated by using organic solvents, such as DMF, THF, and DMAc. And the use of these toxic solvents not only brings serious environmental pollution problems but also poses a great threat to human health [21,22,23]. Recently, Zhou et al. [24] used ethanol/diacetone alcohol as a green solvent to prepare polyamide/polydimethylsiloxane (PA/PDMS) nanofibers for waterproof and breathable membranes. However, according to the GlaxoSmithKline (GSK) [25] solvent selection guidelines, water is the preferred green and pollution-free solvent. Zhou et al. [1] prepared water-based electrospinning nanofiber WBMs using WPU doped with polycarbodiimide (PCD) and long-chain alkyl polymer (LAP) in situ, but the hydrostatic pressure (35.9 kPa) and mechanical properties (2.3 MPa) need to be further improved. Therefore, it is still a challenge to develop a waterborne and elastic nanofibrous membrane with waterproofness and breathability.

In previous studies, we successfully prepared waterborne silicone-modified polyurethane (PUSX) nanofibers for the first time using electrospinning technology. We also investigated the effects of different molecular weights of poly(ethylene oxide) (PEO) and four crosslinking agents on the morphology and physical properties of the nanofibers [26]. Considering the excellent water solubility of PEO as a template polymer, its reactivity with crosslinking agents, and the dual enhancement of water resistance and mechanical properties mediated by the polycarbodiimide (PCC) crosslinking agent through the crosslinking of carboxyl and hydroxyl groups, we expect to explore the potential application of PUSX nanofibrous membranes in waterproof and breathable membranes. In this study, we attempted to utilize a small amount of ultra-high-molecular-weight PEO as a template polymer and in situ dope with a PCC crosslinking agent to fabricate PUSX nanofibrous membranes via a waterborne emulsion electrospinning technique. Through thermal crosslinking and hot-pressing processes, we aimed to form a three-dimensional interconnected crosslinked network structure with smaller pore sizes, endowing the nanofibrous membranes with excellent waterproof and breathable properties. By characterizing the morphology, mechanical properties, waterproofness, air permeability, and water vapor transmission rate, we evaluated the potential applications of PUSX nanofibrous membranes in the field of waterproof and breathable membranes.

## 2. Materials and Methods

### 2.1. Materials

The waterborne silicone-modified polyurethane (PUSX, solid content: 30 wt%), polyurethane (PU, solid content: 30 wt%), and waterborne polycarbodiimide crosslinking agent (PCC) were provided by Shin-Etsu Chemical Industry Co., Ltd. (Tokyo, Japan) and Dainichiseika Color & Chemicals Mfg. Co., Ltd. (Tokyo, Japan). PEO (Mv~8,000,000) was provided by Sigma-Aldrich Co. LLC (Burlington, MA, USA). The above chemicals were used without further purification.

### 2.2. Preparation of Spinning Solutions

The PUSX/PEO spinning solution was prepared by dissolving PEO powder in a PUSX dispersion with mixing and stirring for 48 h at room temperature. The contents of PEO were 0.2, 0.3, 0.4, and 0.5 wt%, respectively. In addition, the content of PEO in the PUSX/PEO/PCC spinning solution was fixed at 0.4 wt%, and the concentration of PCC maintained a gradient increase relative to the solid content of PUSX (5, 10, 15, 20 wt%). The spinning solution was prepared by adding PCC 8–12 h before electrospinning to ensure adequate mixing.

### 2.3. Fabrication of Fluorine-Free Waterborne PUSX Nanofibrous Membranes

The PUSX nanofibrous membrane was prepared by an NEU nanofiber electrospinning device (Kyoto Kato Technology Co., Ltd., Kyoto, Japan). The electrospinning solution was loaded into a 10 mL plastic syringe with a metal needle (18 G, 1.2 mm) and pumped out at a constant rate of 0.1–0.15 mm/min. A high voltage of 18–20 kV was applied to the needle, and the spinning distance was 18 cm. During the electrospinning process, the temperature and relative humidity were 23 ± 2 °C and 50% RH, respectively. The electrospinning time was 5–8 h, and the thickness of the membrane was 80–120 µm. The obtained nanofibrous membranes were heated at 150 °C for 10 min to promote the crosslinking reaction. In addition, the prepared waterborne electrospinning (abbreviated as WE) nanofibrous membranes with different contents of PEO are denoted as WE PUSX-x, where x is 0.2, 0.3, 0.4, and 0.5, representing the increase in the PEO concentration from 0.2 to 0.4 wt%. The nanofibrous membranes with various contents of PCC are represented as WE PUSX-C-y, where y represents 5, 10, 15, and 20, corresponding to PCC crosslinking agent concentrations ranging from 5 to 20 wt%. The hot-pressed nanofibers are represented as WE PUSX-CP-0, WE PUSX-CP-1, WE PUSX-CP-2, and WE PUSX-CP-3, where 0, 1, 2, and 3 denote the hot-pressing temperatures of room temperature (RT), 100, 120, and 140 °C, respectively. Therefore, in the abbreviation ‘WE PUSX-CP’, ‘WE’ stands for ‘waterborne electrospinning’, ‘C’ represents crosslinking, and ‘P’ denotes hot pressing as shown in Table 1.

### 2.4. Characterization

The spinning solution’s viscosity and conductivity were measured using an SV tuning fork vibration viscometer and a personal conductivity meter (SC72, Yokogawa, Tokyo, Japan). The morphology of the nanofibrous membrane was characterized using a scanning electron microscope (JSM-6010LA, JEOL, Tokyo, Japan). The diameter of the nanofibers was measured using Image J 1.5.3e software. The porosity of the membrane was characterized using the liquid displacement method [1,27] using n-hexane as the displacement liquid. The porosity was calculated using the following formula:

$$porosity\;(\%)=\frac{V_{1}-V_{3}}{V_{2}-V_{3}}\times 100$$

where *V*_1_ and *V*_2_ indicate the volume of hexane before and after immersion of the membrane, respectively, and *V*_3_ represents the volume after removal of the nanofibrous membrane. The pore size and distribution of nanofibrous membranes were characterized using a capillary flow porometer (CFP-1200AI, PMI, Newtown Square, PA, USA) at a pressure of 100 kPa. The surface wettability of the samples was tested by dropping 2 µL of pure water on the sample surface using an automated contact angle meter (DMs-400, Kyowa, Niiza, Japan). A small desktop universal tensile tester (EZTest/EZ-S, Shimadzu, Kyoto, Japan) was used to test the mechanical properties of different samples at a tensile speed of 10 mm/min. Mechanical properties during the tensile loading–unloading cycle were evaluated using a tensile testing machine (RTC-1250A, A & D Co. Ltd., Tokyo, Japan) at a rate of 30 mm/min. The area under the stress–strain curve was used to determine toughness (fracture energy) [28]. In addition, the elasticity (strain recovery) of the membrane was calculated using the following formula in the cyclic tensile test [29]:

$$elasticity\;(\%)=\frac{\varepsilon_{m}-\varepsilon_{p}\left(N\right)}{\varepsilon_{m}}\times 100$$

where $\varepsilon_{m}$ represents the maximum strain (400%) of the nanofibrous membrane, and $\varepsilon_{p}\left(N\right)$ represents the residual strain after the Nth unloading. The structures and surface elements of the samples were characterized via attenuated total reflection-Fourier transform infrared spectroscopy (FT/IR-6600•IRT-5200, Jasco, Hachioji, Japan), X-ray photoelectron spectroscopy (XPS, AXIS-ULTRA HSA SV, Shimadzu Corporation, Kyoto, Japan), and energy dispersion spectroscopy (EDS, JEOL, Tokyo, Japan). The washability of the WE PUSX-CP-2 membrane was tested using the AS ONE ultrasonic cleaning machine (US-1R, AS ONE Corporation, Osaka, Japan) at a frequency of 40 kHz. Cut the membrane into a rectangular shape of 2 × 8 cm, immerse it in deionized water, sonicate for 1 h, then remove and dry it, which is defined as a washing cycle. Ultrasonic washing was performed for 24 cycles, and the water contact angle (WCA) was measured after each cycle [30].

### 2.5. Characterization of Waterproofness and Breathability

Hydrostatic pressure tests were conducted using a water permeability tester (YG812F, Wenzhou Fangyuan Instrument Co., Ltd., Wenzhou, China) at a constant rate of a water pressure increase of 6 kPa min^−1^. The pressure area was 100 cm^2^. A piece of plain fabric was placed on the membrane surface to prevent excessive deformation during the test. The water vapor transmission rate tester (W3/031, Jinan Languang Electromechanical Technology Co., Ltd., Jinan, China) was used, following the standards of Method A (positive cup method) in GB/T 12704-1991 [31], the testing environment was maintained at a temperature of 38 °C and 90% relative humidity, and the value of the WVT rate was calculated using the following formula:

(1) $$WVT=\frac{24\cdot ∆m}{S\cdot t}$$

where the *WVT* rate stands for the water vapor transmission per square meter tested for one day, g m^−2^d^−1^. ∆*m* refers to the weight difference before and after the water vapor transmission cup, g. *S* represents the test area, m^2^. *t* indicates the testing time, day. The diameter of the test sample was 8 cm. In addition, the air permeability of the nanofibrous membrane was evaluated using a fully automatic permeability meter (YG461E-III, Ningbo Textile Instrument Factory, Ningbo, China) at a pressure difference of 100 Pa.

## 3. Results and Discussion

### 3.1. Design of Environmentally Friendly PUSX Nanofibrous WBMs via Waterborne Electrospinning

**Figure 1:**
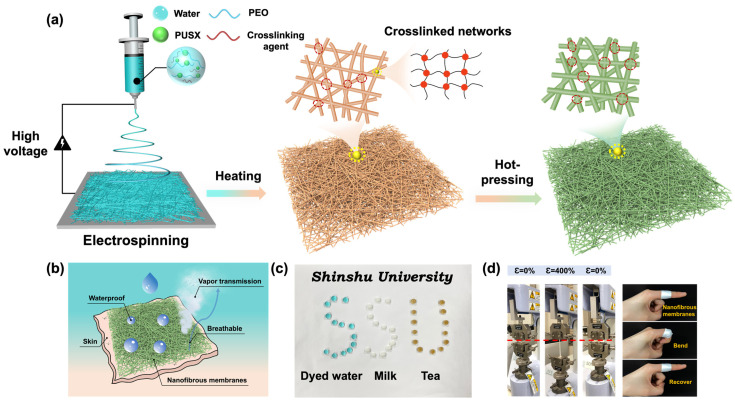
(**a**) Schematic of the formation process of waterborne silicone-modified polyurethane nanofibrous membranes with hydrophobicity and an interconnected porous structure. (**b**) Schematic representation of the waterproof and breathable properties of the membrane. The photographs demonstrate the (**c**) resistance to liquid water and (**d**) mechanical properties of WE PUSX-CP-NFMs.

We prepared green WBMs with excellent waterproof and breathable properties based on the following principles: (1) achieving good hydrophobicity without the use of fluorinated compounds; (2) the preparation process of nanofibrous membranes should be green and environmentally friendly, without the use of toxic solvents; (3) WBMs should have high porosity and interconnecting channels to ensure good breathability, as well as small pore sizes to improve waterproofness. To satisfy the above three principles, we used a waterborne silicone-modified polyurethane (PUSX) dispersion with a molecular formula as shown in Scheme S1. In this process, silicone endows the PUSX dispersion with hydrophobicity. In previous studies, the fiberization of PUSX was successfully achieved by blending PUSX with PEO and the in situ doping of a crosslinking agent [26]. In this study, we attempted to prepare high-performance fluorine-free elastic waterproof and breathable nanofibrous membranes by adding a small amount of ultra-high-molecular-weight PEO as a template polymer and in situ doping the PCC crosslinking agent, as shown in Figure 1a. Then, via simple heating treatment, the carbodiimide groups (-N=C=N-) in PCC undergo a reaction with the carboxyl groups (-COOH) in PUSX to construct a network. Meanwhile, the hot-pressing treatment can decrease the pore size to prevent liquid water from penetrating while also promoting the migration and enrichment of hydrophobic silyl groups on the fiber surface, while reducing the pore size to resist the penetration of liquid water. Figure 1b shows a schematic diagram of the waterproofness, air permeability, and moisture permeability of the nanofibrous membrane. The waterborne electrospinning PUSX composite nanofibrous membrane prepared in this study has a highly interconnected network structure that allows air and water vapor to penetrate and resist the penetration of liquid water (such as water, milk, and tea, etc.), as shown in Figure 1c. In Figure 1d, we demonstrate that under a super strong tensile strain of 400%, the nanofibrous membrane can easily restore its shape, even after being forcefully bent and stretched by fingers. In the stretching experiment, the red dashed line indicates the starting position of the stretch. Therefore, this simple and environmentally friendly preparation method provides a highly promising choice for eco-friendly nanofiber WBMs, which is expected to be widely used in various fields, including outdoor clothing, healthcare, water purification, and wearable electronic devices.

### 3.2. Characterization of Waterborne PUSX Dispersion

**Figure 2:**
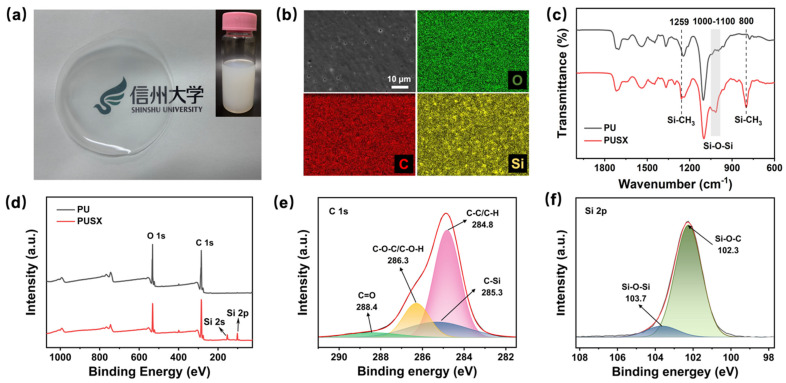
(**a**) Digital photograph of PUSX film, illustration showing PUSX dispersion. (**b**) SEM image and EDS mapping image of PUSX film. (**c**) FTIR and (**d**) XPS spectra of PU and PUSX films. XPS high-resolution (**e**) C1s and (**f**) Si 2p spectra of PUSX films.

We synthesized an anionic self-emulsifying waterborne silicone-modified polyurethane (PUSX) dispersion with a solid content of 30 wt% (inset of Figure 2a) using the pre-polymer method using poly(tetramethylene glycol) (Poly THF 2000), 4,4-methylenebis (cyclohexyl isocyanate) (H12MDI), a bipartite organosilicone glycol, and the aqueous dispersing agent dihydroxymethylpropionic acid (DMPA). The molecular structure and synthesis process of PUSX are shown in Schemes S1 and S2. The PUSX dispersion stood at room temperature for 3 months, as shown in Figure S1, indicating its excellent stability. The cast film obtained by evaporating the water from the PUSX dispersion is shown in Figure 2a, and the cast film exhibits a clearer semi-transparent shape. As shown in Figure 2b, from the SEM and EDS mapping images, silicone was detected on the surface of the PUSX film without the presence of harmful fluorine elements that are not easily degraded. And no silicone element was detected on the surface of the PU film (Figure S2a). The elemental content of PUSX and PU films is shown in Figure S2b,c. From the FTIR spectrum in Figure 2c, it can be seen that in the spectrum of PUSX, the stretching peaks of Si-C appeared at 800 cm^−1^ and 1259 cm^−1^, and the stretching vibration peak belonging to Si-O-Si also appeared at 1053 cm^−1^ [32,33,34]. This indicates that silicone has been synthesized into the chain segment of polyurethane. XPS was used to study the surface elements and bonding configurations of the films. As shown in Figure 2d, the PU and PUSX films had the same oxygen (532.1 eV for O1s) and carbon (284.8 eV for C1s) characteristic peaks [17], but the Si 2s and Si 2p characteristic peaks belonging to silicon appeared in the spectra of PUSX. Figure 2e shows that the C 1s of the PUSX film was deconvoluted into four peaks with binding energies (BE) at 288.4 eV, 286.3 eV, 284.8 eV, and 285.3 eV, which corresponded to C=O, C-O-C/C-O-H, C-C/C-H, and C-Si peaks, respectively [30,35,36]. As shown in Figure 2f, the high-resolution scanning spectra of Si 2p were fitted with characteristic peaks belonging to Si-O-Si (103.7 eV) and Si-O-C (102.3 eV), which further proved that PUSX had been synthesized successfully [37,38].

### 3.3. Formation and Structural Stability of Waterborne Electrospun PUSX Nanofibers

**Figure 3:**
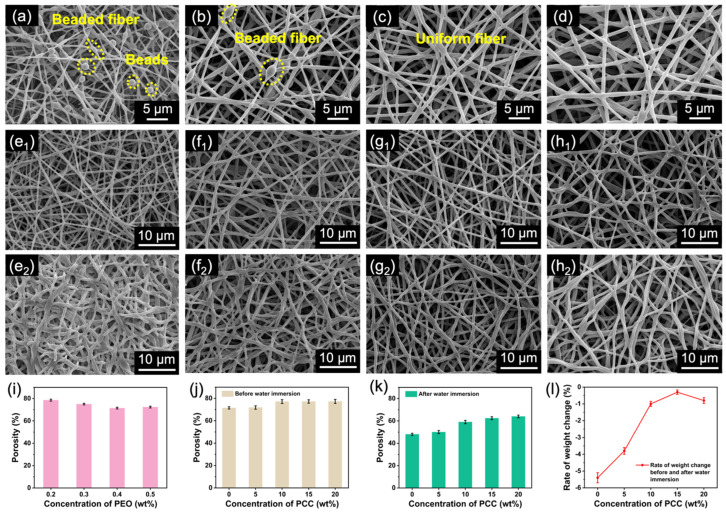
SEM images of WE PUSX membranes obtained from PEO solutions with concentrations of (**a**) 0.2, (**b**) 0.3, (**c**) 0.4, and (**d**) 0.5 wt%. SEM images of WE PUSX-C with different contents of PCC (5, 10, 15, and 20 wt%) before (**e_1_**–**h_1_**) and after (**e_2_**–**h_2_**) immersion in water. The porosity of (**i**) WE PUSX membranes, (**j**) before water immersion of WE PUSX-C membranes and (**k**) after water immersion of WE PUSX-C membranes. (**l**) The rate of weight change before and after water immersion with various PCC concentrations.

**Figure 4:**
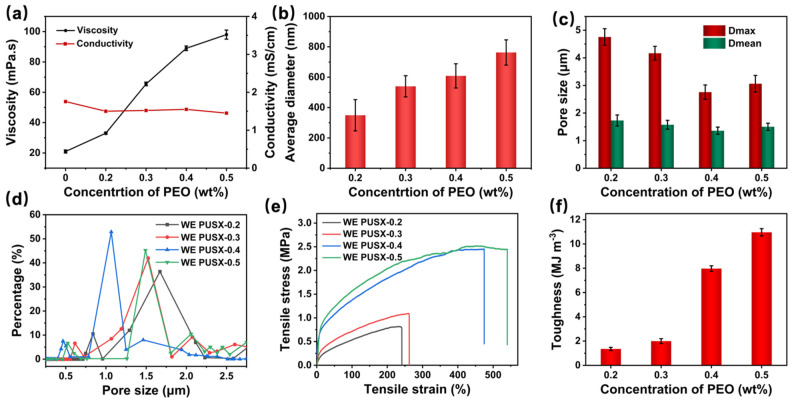
The change, with different PEO concentrations, in (**a**) viscosity and conductivity, (**b**) mean diameter, and (**c**) D_max_ and D_mean_, and the (**d**) pore size distribution, (**e**) tensile stress–strain curves, and (**f**) toughness.

Waterborne PUSX dispersions have short molecular chains, low viscosity, and a lack of sufficient entanglement between molecular chains. Therefore, they are difficult to stretch into continuous fibers during electrospinning and can only form a bead structure (Figure S3) [39]. In this study, we selected PEO with a higher molecular weight as a template polymer to increase the entanglement of molecular chains in the spinning solution and enable it to be stretched and formed in electrospinning. We prepared waterborne electrospun PUSX (WE PUSX) nanofibrous membranes with different PEO contents and studied the effect of the PEO content on the morphology and properties of membranes. As shown in Figure 3a–d, the addition of a small amount of PEO (0.2–0.5 wt%) can increase the viscosity of the PUSX spinning solution (Figure 4a), which drives the spinning formation of PUSX. And as the PEO content increased, the WE PUSX nanofibrous membrane gradually changed from bead and beaded fibers (Figure 3a,b) to uniform nanofibers (Figure 3c,d), which was mainly attributed to the sufficient entanglement of PUSX and PEO molecular chains. Moreover, there was a certain adhesion between adjacent fibers, which also increased the waterproof performance of the WE PUSX membrane to some extent. As shown in Figure 4b, the diameter of the nanofibers gradually increased from 349 nm to 763 nm with the increase in the PEO concentration. This can be attributed to the increased viscosity caused by the increased PEO content, although it had little effect on the conductivity (Figure 4a). As the PUSX nanofibrous membrane changed from beaded fibers to uniform fibers, the pore size of the nanofibrous membrane gradually decreased (Figure 4c), and the pore size distribution shifted to the left (Figure 4d), resulting in a decrease in the porosity of the nanofibrous membrane (Figure 3i). When the concentration of PEO was increased to 0.5 wt%, the pore size and porosity of the nanofibrous membrane also slightly increased, which could be attributed to the larger nanofiber diameter leading to an increase in the inter-fiber space. Considering that larger pore sizes would depress the waterproof performance, the optimal concentration of PEO was determined to be 0.4 wt%.

To enhance the water resistance of PUSX nanofibrous membrane and build a stable porous structure, we constructed a crosslinked network via the in situ doping of PCC [26]. As shown in Figure 3e_1_–h_1_, SEM images of nanofibrous membranes with different PCC concentrations showed a smooth and uniform fiber morphology, and the porosity of the fibrous membrane (Figure 3j) also increased with the increase in the PCC content. This may be due to the crosslinking network fixing the position of nanofibers, preventing fiber-to-fiber movement, and increasing the gaps between fibers. The adhesion between the membrane fibers after the addition of PCC still exists, but this adhesion has transformed into chemical adhesion due to the occurrence of crosslinking reactions. Moreover, the excessive content of a crosslinking agent leads to excessive crosslinking between nanofibers or self-reaction of the crosslinking agent, which in macroscopic terms showed a significant increase in adhesion between nanofibers (Figure 3h_1_).

In addition, the fiber morphology after immersion is shown in Figure 3e_2_–h_2_. Nanofibrous membranes with a low crosslinking agent concentration were very sensitive to water treatment, and fusion occurred between adjacent fibers, resulting in a low porosity of only 50%. As the content of the crosslinking agent increased, the porous structure of the prepared waterborne electrospun PUSX crosslinked (WE PUSX-C) nanofibrous membrane became increasingly stable. The rate of weight change before and after immersion also significantly decreased (Figure 3l). After water immersion, the WE PUSX-C-15 and WE PUSX-C-20 membranes can maintain a clear nanofiber morphology. Correspondingly, the porosity of the nanofibrous membrane has significantly improved, with an average pore size of 1.38 µm and 1.5 µm, respectively (Figure S4). As the content of PCC increased from 0 wt% to 20 wt%, the porosity of the WE PUSX-C nanofibrous membrane after water treatment increased from 48% to 64%. This indicates that as the crosslinking agent increases, the number of crosslinking points increases, and the structure of the nanofibrous membrane becomes more stable, as shown in Figure 3k. However, the further increase in PCC will lead to uneven and thicker nanofibers (Figure S5), potentially attributable to excessive crosslinking or self-reaction crosslinking caused by the higher concentration of the crosslinking agent.

**Figure 5:**
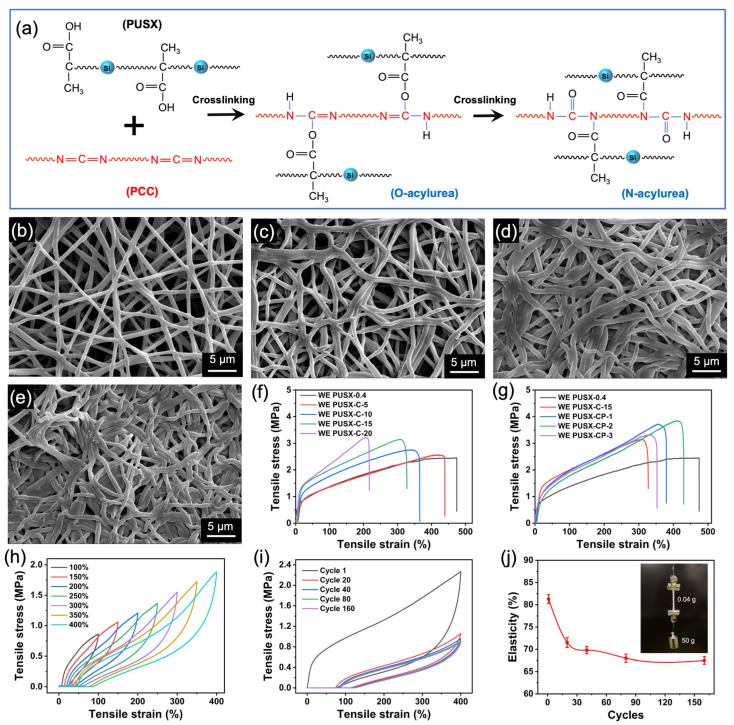
(**a**) Crosslinking reaction formula of PUSX and PCC. SEM images showed the surface morphology and structures of WE PUSX-C-15 under hot-pressing at (**b**) room temperature, (**c**) 100 °C, (**d**) 120 °C, and (**e**) 140 °C at a pressure of 10 MPa. Stress–strain curves at different concentrations of the crosslinking agent (**f**,**g**) under different hot-pressing conditions. (**h**) The WE PUSX-CP-2 membranes tensile loading and unloading stress–strain curves at strains of 100, 200, 300, and 400%, and (**i**) cyclic tensile stress–strain curves with a strain of 400%. (**j**) Variation in elasticity with the number of cycles.

Figure 5a shows the chemical reaction mechanism of the crosslinking agent. Firstly, the carbodiimide group (-N=C=N-) in PCC activates the carboxyl group (-COOH) to generate the intermediate O-acylurea. Further, through internal rearrangement or a reaction with another -COOH, the carbonyl group is transferred from the oxygen atom to the adjacent nitrogen atom, generating N-acylurea [40]. Microscopically, the generation of crosslinking points caused molecular chains to change from short chains to long chains, forming a three-dimensional interconnection network. Macroscopically, there was adhesion between the crosslinked fibers. In addition, excessive crosslinking agents caused excessive crosslinking between fibers or self-reaction crosslinking, leading to increased internal stress and ultimately deformation of the fibers (as shown in Figure 3h_1_). Additionally, the influence of the crosslinking temperature on the crosslinking behavior was explored in Figure S6, and the optimal temperature was determined to be 150 °C for 10 min. We analyzed the chemical structure of nanofibrous membranes using FTIR spectroscopy. As shown in Figure S7, an absorption peak belonging to the PCC functional group N=C=N appeared at 2118 cm^−1^. As the PCC content increased, the absorption peaks in the infrared spectrum at 3330 cm^−1^ (corresponding to the -NH stretching vibration), 1717 cm^−1^ (related to the C=O stretching vibration), and at 1641, 1545, and 1237 cm^−1^ (representing amide bond I, II, and III bands) gradually intensified. These changes indicate that more -COOH groups reacted with N=C=N, leading to the formation of N-acyl urea groups and the construction of a crosslinked network [41,42,43]. The FTIR sample of WE PUSX-C-15 showed that N=C=N had almost completely reacted without any obvious excess, indicating that all carboxyl groups had participated in the crosslinking reaction. However, the WE PUSX-C-20 exhibited a very strong N=C=N characteristic peak, which may be attributed to the excessive addition of PCC that did not participate in the crosslinking reaction.

The schematic diagram of the formation mechanism of WE PUSX-C nanofibrous membrane is shown in Figure S8. The PEO molecules and PUSX molecules are randomly distributed in the spinning solution. During the one-step electrospinning process, under the action of high voltage, the PUSX molecular chains gradually fold and orient with the PEO long chain [44]. Continuous and uniform nanofibers will be produced following the stretching, aggregation, and demulsification process when the concentration of the PUSX/PEO/PCC mixed spinning solution surpasses the entanglement concentration [12,45].

### 3.4. Improvement of Mechanical Properties of WE PUSX Nanofibrous Membrane with Crosslinking and Hot-Pressing

Mechanical properties play an important role in practical applications. When the content of the template polymer PEO reached 0.4 wt%, the tensile stress (2.45 MPa), strain at break (473.7%), and toughness (7.97 MJ m^−3^) of PUSX nanofibrous membranes were significantly improved, as shown in Figure 4e,f. This is attributed to the disappearance of beads, beaded fibers, and the formation of uniform fibers. When the content of PEO increased to 0.5 wt%, the larger fiber diameter led to a stronger individual fiber, and a slight increase in the mechanical properties of the membrane could be observed (Figure 4e,f). Moreover, the D_max_ of the nanofibrous membrane begins to increase at this time, which may be due to the accumulation of thicker fibers leading to the formation of larger fiber gaps between adjacent fibers. Considering the pore structure, water resistance, and mechanical properties of the nanofibrous membrane, the concentration of 0.4 wt% PEO was finally selected to explore the influence of the subsequent crosslinking agent content.

The tensile mechanical properties of the WE PUSX-C nanofibrous membrane are shown in Figure 5f and Figure S9. As the content of the crosslinking agent increased, the tensile strength gradually increased, which was different from the previous report that crosslinking agents can reduce the tensile strength of WPU [1]. This may be related to the doping of silicone chains [46,47,48]. The elongation at break gradually decreased with the increase in the PCC concentration, which can be attributed to the increase in crosslinking points and the increase in rigidity of the nanofibrous membrane leading to a decrease in flexibility. When the concentration of PCC was 20 wt%, the toughness decreased sharply and the fracture strain decreased to 221%, but the increase in stress was not significant, which may be the result of excessive crosslinking, as shown in Figure 3h_1_ earlier. Considering the mechanical properties and flexibility required by textile fabrics in practical applications, we have chosen WE PUSX-C-15 as the optimal sample.

Hot-pressing treatment can regulate the pore size of nanofibrous membranes and enhance their mechanical properties [49,50]. We subjected the WE PUSX-C-15 membrane to hot-pressing treatment at temperatures of 100, 120, and 140 °C for 5 s at a pressure of 10 MPa, respectively. The morphology of the nanofibrous membrane after hot-pressing is shown in Figure 5b–e. This indicated that as the temperature increased, the pore size of the membrane decreased, and adhesion occurred between adjacent fibers. Figure S10 shows the mechanism diagram of hot-pressing treatment, which causes the fibers to transform from cylindrical to elliptical cylinders, with larger diameters and smaller pore sizes. When the hot-pressing temperature increased from 100 °C to 140 °C, the maximum pore size of the nanofibrous membrane decreased from 2.85 μm to 1.9 μm, and the porosity decreased from 66% to 31% (Figure S11a). The pore size distribution in Figure S11b reveals that as the hot-pressing temperature increased, the average pore size of the nanofibrous membrane decreased, which is advantageous for improving its waterproofing performance. The membrane after hot-pressing exhibited a uniform pore size. The mechanical properties of the hot-pressed sample and the original sample are shown in Figure 5g and Figure S11c. The stress–strain curves showed that, compared to the pure WE PUSX samples, the nanofibrous membrane (WE PUSX-C) with the addition of PCC exhibited a reduction in fracture strain and toughness, while enhancing the tensile stress. Hot-pressing treatment reduced the pore size while increasing the mechanical properties of the nanofibrous membrane. When the hot-pressing temperature was 120 °C, the tensile stress and toughness of the WE PUSX-CP-2 nanofibrous membrane showed their maximum values, which were 3.85 MPa and 10.37 MJ m^−3^, respectively.

To simulate the ability of nanofibrous membranes to resist external deformation in practical conditions, we further investigated the cyclic loading–unloading tensile behavior of WE PUSX-CP-2 membranes. As shown in Figure 5h and Figure S12, the nanofibrous membrane exhibited amazing stretchability and ultra-high elasticity (~80%) under different strains of 100~400%. Moreover, the nanofibrous membrane was repeatedly stretched at an extremely high tensile strain of 400% without damage. As shown in Figure 5i,j, it still exhibited ultra-high elasticity (~65%) after 160 cycles of stretching, exhibiting impressive stretchability. This may be attributed to the introduction of silicone groups leading to chemical reactions with polyurethane molecular chains, thereby increasing the elasticity of the nanofibrous membrane, or due to the inherent durability of silane groups, the nanofibrous membrane can quickly recover to its original shape when subjected to external forces. In addition, the nanofibrous membrane with a weight of 0.04 g can easily withstand a 50 g weight (illustrated in Figure 5j).

### 3.5. Waterproof and Breathable Properties

**Figure 6:**
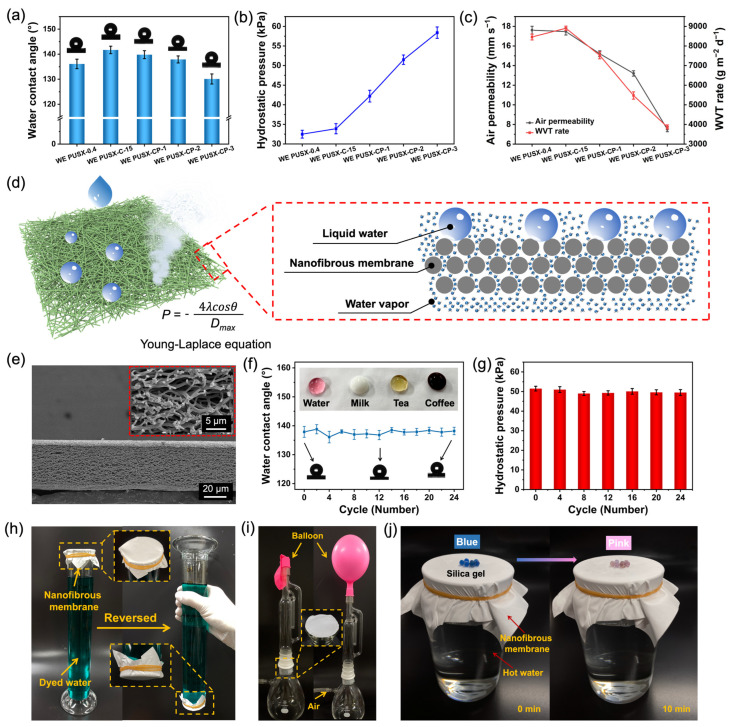
(**a**) WCA, (**b**) hydrostatic pressure, and (**c**) air permeability and WVT rate of the membranes. (**d**) Schematic mechanism of waterproof and breathable properties of the membranes. (**e**) Cross-section view of WE PUSX-CP-2 nanofibrous membranes (the inset is a high-magnification cross-section view). The change in (**f**) WCA and (**g**) hydrostatic pressure with different washing times. The photographs demonstrate the (**h**) waterproof, (**i**) air permeable, and (**j**) water vapor transmission properties of WE PUSX-CP-2 membranes.

As shown in Figure 6a, the water contact angle (WCA) was used to characterize the surface wettability of the nanofibrous membrane. The good WCA (136.1°) of the WE PUSX-0.4 nanofibrous membrane was shown due to the presence of silicone and the porous structure of nanofibers. The WCA of the WE PUSX-C-15 nanofibrous membrane was further increased to 141.7° due to the crosslinking of hydrophilic groups in PUSX. After hot-pressing the WE PUSX-C-15 nanofibrous membrane at room temperature, 100, 120, and 140 °C, the WCA of the nanofibrous membrane was 141.1°, 139.8°, 137.9°, and 130.1°, respectively. The decrease in the contact angle can be attributed to the reduction in the surface roughness of nanofibers caused by hot pressing. From the results of hydrostatic pressure (Figure 6b), it can be observed that when the hot-pressing temperature was higher than room temperature, the hydrostatic pressure of the nanofibrous membrane increased rapidly. This may be attributed to the closure of larger pores and narrowing of the pore size distribution (Figure S11a,b), while the change in WCA was not significant. When the hot-pressing temperature reached 140 °C, the hydrostatic pressure of the nanofibrous membrane showed an astonishing 58.4 kPa. This can be explained by Laplace’s law. The waterproof and breathable mechanism of the microporous membrane is shown in Figure 6d. Liquid water always prioritizes passing through larger pores, and water resistance is usually used to evaluate waterproofing performance. Using Laplace’s law to explain the waterproofing mechanism, the following is obtained:

(2) $$P=-\frac{4\lambda cos\theta }{D_{max}}$$

where *P* represents the hydrostatic pressure, *λ* represents the surface tension of water, *θ* represents the water contact angle of the membrane, and *D_max_* represents the maximum pore size of the membrane. Therefore, the water resistance of electrostatic spinning nanofibrous membranes is mainly attributable to their high surface hydrophobicity and small pore size.

Breathability plays an important role in textile fabrics. Good breathability can promote heat removal from the body and increase skin comfort. We evaluated the breathability of nanofibrous membranes based on air permeability and moisture permeability. As shown in Figure 6c, the air permeability and water vapor transmission rate of the nanofibrous membrane exhibited a decreasing trend with an increase in the hot-pressing temperature. This is because of the increase in fusion between adjacent fibers at high temperatures, which reduces or closes part of the pore size of the nanofibrous membrane and decreases the porosity (Figure S11a). The cross-sectional view of the WE PUSX-CP-2 nanofibrous membrane is shown in Figure 6e (inset is the corresponding high-magnification image). Despite undergoing hot-pressing treatment, the membranes retained many interconnected channels. This may be due to the inherent loose structure of the nanofibers, which was insufficient to completely melt or fuse all fibers after a hot-pressing treatment of only 5 s and at a lower temperature. Consequently, some fibers, except those on the surface, retained their original structure, leading to the formation of these interconnected channels. These interconnected channels facilitate the transfer of air and water vapor. The WE PUSX-CP-3 nanofibrous membrane has the highest hydrostatic pressure, but the WVT decreases to 3875 g m^−2^ d^−1^, which is not conducive to heat diffusion and affects the comfort of waterproof and breathable fabrics. Considering the waterproofness and breathability, we have ultimately chosen the WE PUSX-CP-2 nanofibrous membrane, which has a hydrostatic pressure of 51.5 kPa, an air permeability of 13.2 mm s^−1^, and a WVT rate of 5476 g m^−2^ d^−1^. The performance comparison of the WE PUSX-CP-2 membranes with other fluorine-free WBMs is shown in Table S1.

As shown in Figure 6f, the durability of the WE PUSX-CP-2 nanofibrous membrane was investigated based on the experiment of ultrasonic washing for 24 h. The findings demonstrated that the WCA of the membrane remained almost unchanged (~137.5°) after long washing, and the washed nanofibrous membrane still exhibited good hydrophobicity for pure milk, tea, and coffee (inset of Figure 6f). Meanwhile, the hydrostatic pressure was almost maintained at ~50 kPa, which also proved the waterproof properties of the membrane (Figure 6g). Good washing resistance and waterproofness are particularly important in practical applications. Figure 6h–j shows a series of self-assembly tests to verify the waterproof and breathable properties of the WE PUSX-CP-2 nanofibrous membrane. Under the gravity of 250 g of water, the fibers did not break or penetrate, demonstrating their excellent water resistance. Placing the balloon in a self-assembled device and introducing air, the air can easily blow up the balloon through the nanofibrous membrane, demonstrating its excellent air permeability. In addition, the WE PUSX-CP-2 nanofibrous membrane was secured over a beaker containing hot water, with silica gel particles placed on its surface. After 10 min, the color of silica gel particles on the membrane changed from blue to pink, proving that water vapor could penetrate the nanofibrous membrane and indicating good moisture permeability.

## 4. Conclusions

We propose a simple, eco-friendly, and fluorine-free approach to fabricate highly elastic, waterproof, and breathable silicone-modified polyurethane (PUSX) nanofibrous membranes using waterborne electrospinning technology. Combined with the heat treatment process, PUSX nanofibrous membranes with exceptional water resistance, breathability, and high elasticity were successfully developed without using toxic organic solvents, offering significant advantages over traditional non-fluorinated waterproof and breathable membrane preparation methods. The crosslinking reaction between the poly(carbodiimide) crosslinking agent (PCC) and the PUSX enabled the formation of a stable three-dimensional interconnected crosslinked network, which gave the nanofibrous membrane a porous structure. The hot-pressing treatment further reduced the size of pores, resulting in the excellent waterproof performance of the membrane. The experimental results showed that the prepared WE PUSX-CP-2 membrane had high hydrophobicity (137.9°), significantly surpassing conventional waterborne polyurethane nanofibers (usually < 90°) [51,52]. The hydrostatic pressure of the membrane was 51.5 kPa, and the WVT rate was 5476 g m^−2^ d^−1^, which was comparable to that of organic solvent-based nanofiber WBMs [19,53,54,55,56]. In addition, the membrane had a good air permeability of 13.2 mm s^−1^, which was much higher than that of the post-coating-treated nanofibrous membrane [4,14,17,18,54]. The tensile strength was 3.85 MPa. After multiple stretching events at a strong tensile strain of 400% (160 cycles), the nanofibrous membrane still maintained excellent high elasticity, with an elasticity of 67.5%, which was much higher than previous research results [1,2,16,19,56,57,58,59,60]. This outstanding waterproof and breathable performance and high elasticity gives it great application potential in many fields, such as protective textiles, medical and health products, oil–water separation, etc., and provides an important guarantee for creating a more comfortable microclimate.

## Figures and Tables

**Table 1:** Sample codes and components of PUSX nanofibrous membranes.

Samples	PEO Concentration (wt%)	PCC Concentration (wt%)	Hot-Pressing Temperature (°C)
WE PUSX-0.2	0.2	-----	-----
WE PUSX-0.3	0.3		
WE PUSX-0.4	0.4		
WE PUSX-0.5	0.5		
WE PUSX-C-5	0.4	5	-----
WE PUSX-C-10		10	
WE PUSX-C-15		15	
WE PUSX-C-20		20	
WE PUSX-CP-0	0.4	15	Room temperature
WE PUSX-CP-1			100
WE PUSX-CP-2			120
WE PUSX-CP-3			140

## Data Availability

Data are contained within the article and Supplementary Materials.

## Supplementary Materials

The following supporting information can be downloaded at: https://www.mdpi.com/article/10.3390/polym16111505/s1, Scheme S1: The molecular structure of the synthesized waterborne PUSX; Scheme S2: Synthesis process of waterborne PUSX; Figure S1: Digital photos of PUSX standing for three months; Figure S2: The SEM image and EDS mapping images of PU casting films; Figure S3: The SEM image of PUSX beads obtained from spinning solution (only PUSX 30 wt%) without PEO; Figure S4: D_max_ and D_mean_ of WE PUSX-C membranes with different PCC content after water immersion; Figure S5: Average diameter of WE PUSX-C membranes with different PCC content after water immersion; Figure S6: SEM images of WE PUSX-C nanofibrous membranes heated at different temperatures of 90, 120, 150, and 180 °C (a_1_–d_1_) before and (a_2_–d_2_) after immersion treatment in water; Figure S7: FTIR spectra of WE PUSX-C membranes with different PCC content; Figure S8: Schematic illustration of the formation of WE PUSX-C nanofibers via emulsion electrospinning; Figure S9: The toughness of WE PUSX-C membranes with different PCC content; Figure S10: Hot-pressing mechanism diagram; Figure S11: (a) pore size and porosity, (b) pore size distribution and (c) toughness of membrane with different hot-pressing temperature; Figure S12: Elasticity of WE PUSX-CP-2 nanofibrous membranes under different tensile strains; Table S1: A comparison of waterproof and breathable performances of WE PUSX-CP-2 membranes with other fluorine-free WBMs. References [61,62] are cited in the supplementary materials.
