# Supplementary material for: Fabrication of Waterborne Silicone-Modified Polyurethane Nanofibers for Nonfluorine Elastic Waterproof and Breathable Membranes
Source: Polymers (Basel). 2024 May 25;16(11):1505. doi: 10.3390/polym16111505 (PMC11174452; doi:10.3390/polym16111505)
Supplement: Supplementary file 1 [file polymers-16-01505-s001.zip › polymers-3026135-supplementary.pdf]

polymers

MDPI

Article

Fabrication of waterborne silicone-modified polyurethane nanofibers for nonfluorine elastic waterproof and breathable membranes

Fang Li [1], Kai Weng [1], Toshihisa Tanaka [1,*], Jianxin He [2], Haimin Zheng [2], Daisuke Noda [3], Shinji Irifune [3] and Hiroshasa Sato [4]

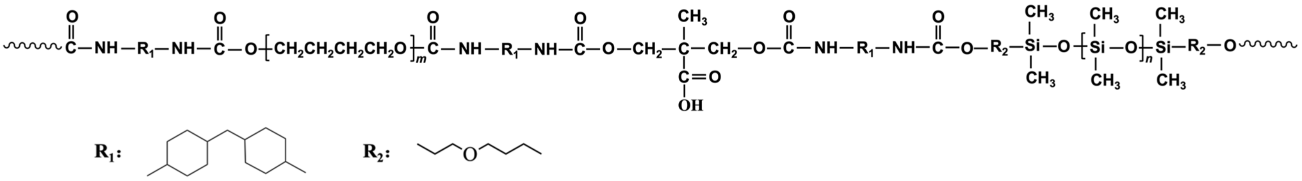

Scheme S1. The molecular structure of the synthesized waterborne PUSX.

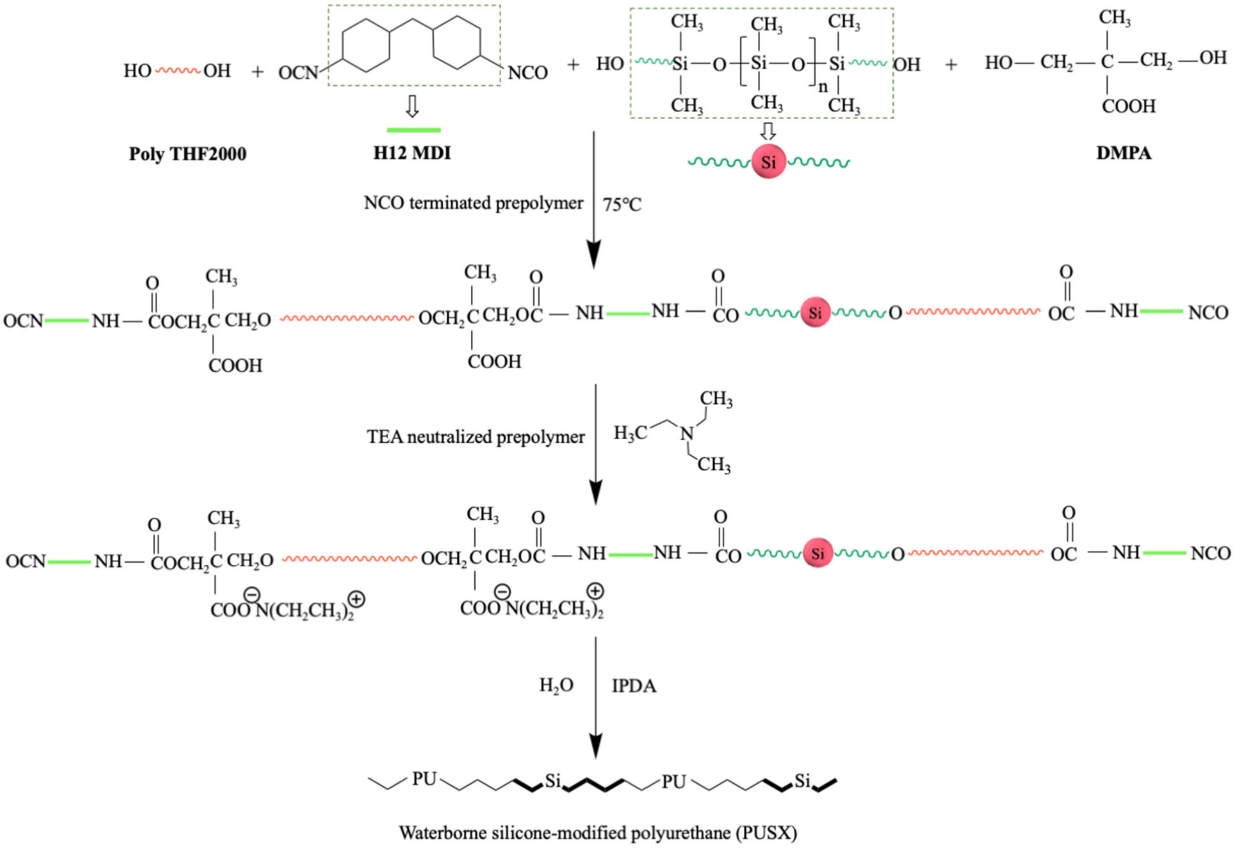

Scheme S2. Synthesis process of waterborne PUSX.

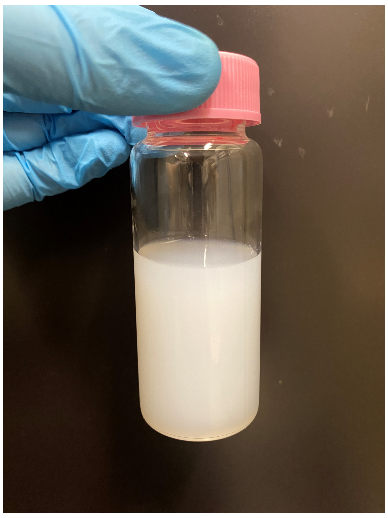

Figure S1. Digital photos of PUSX standing for three months. This indicates that it has good stability.

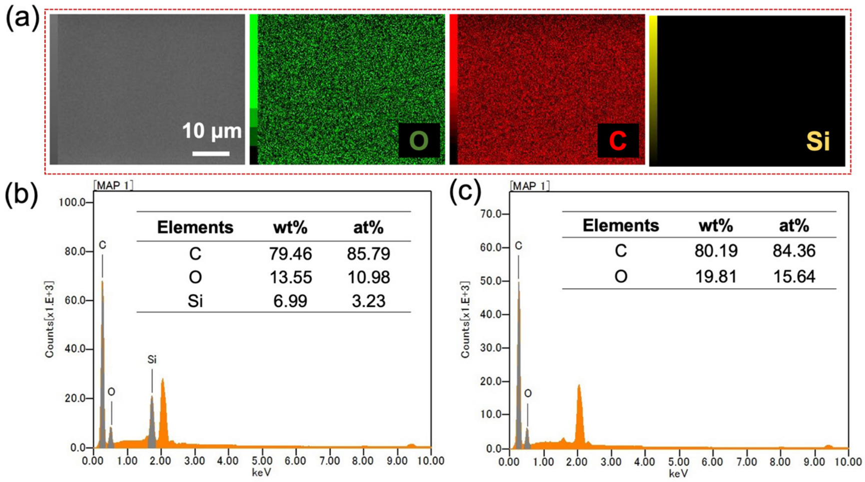

Figure S2. (a) The SEM image and EDS mapping images of PU casting films. The distribution and content of elements in (b) PUSX and (c) PU films.

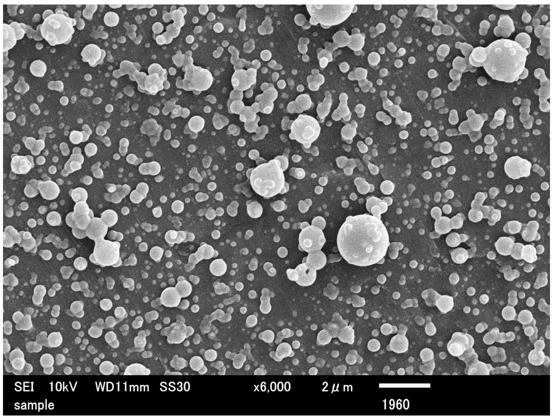

Figure S3. The SEM image of PUSX beads obtained from spinning solution (only PUSX 30 wt%) without PEO.

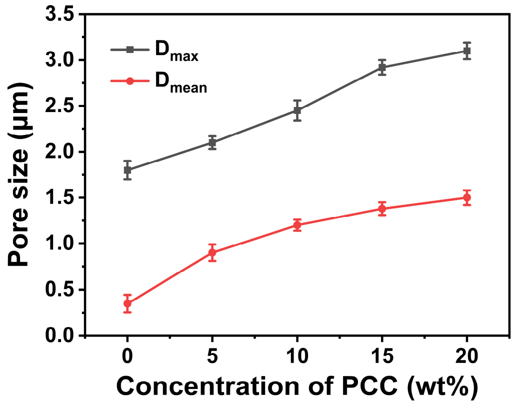

Figure S4. D_{max} and D_{mean} of WE PUSX-C membranes with different PCC content after water immersion.

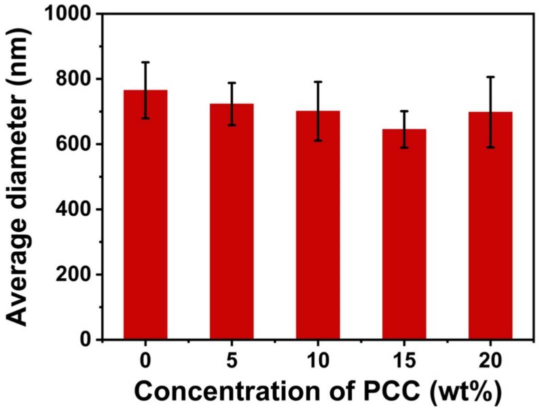

Figure S5. Average diameter of WE PUSX-C membranes with different PCC content after water immersion.

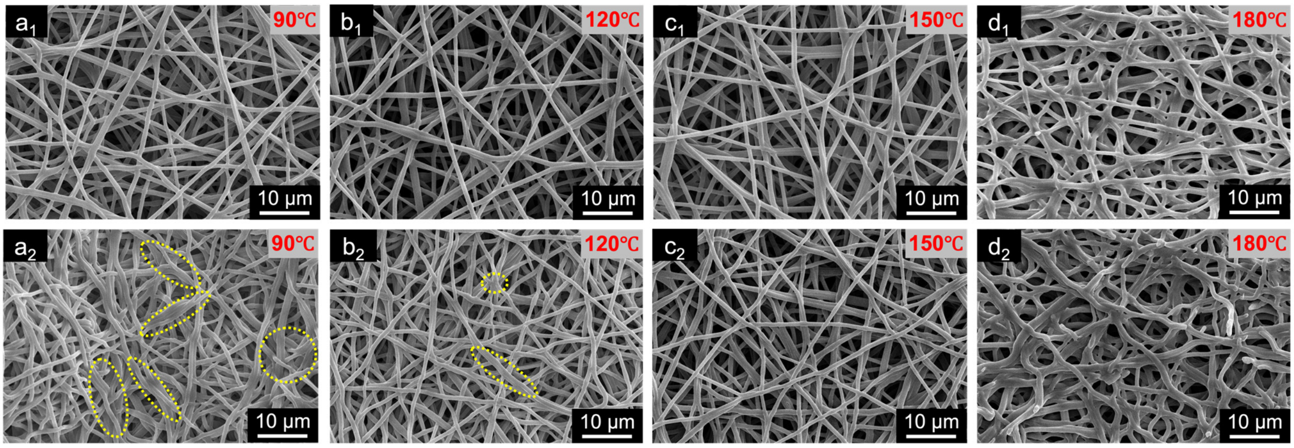

Figure S6. SEM images of WE PUSX-C nanofibrous membranes heated at different temperatures of 90, 120, 150, and 180℃ (**a_1**-**d_1**) before and (**a_2**-**d_2**) after immersion treatment in water. The WE PUSX-C-15 membranes were heat-treated at 90, 120, 150, and 180℃ for 10 min, immersed in deionized water for 24 h, and dried at room temperature. From Fig. S6a_2, it can be observed that samples crosslinked at a low temperature of 90℃ for 10 minutes exhibited sensitivity to water treatment, with severe fiber adhesion observed between fibers. Nanofibrous membranes crosslinked at 120℃ displayed a relatively clear morphology after water treatment, but still had a small amount of adhesion. The membrane crosslinked at 150℃ maintained a clear fiber morphology after water treatment. However, excessive crosslinking temperature (180℃) disrupted the fiber structure,

resulting in severe fusion between adjacent fibers. Therefore, we selected 150℃ and 10 min as the crosslinking temperature for PCC.

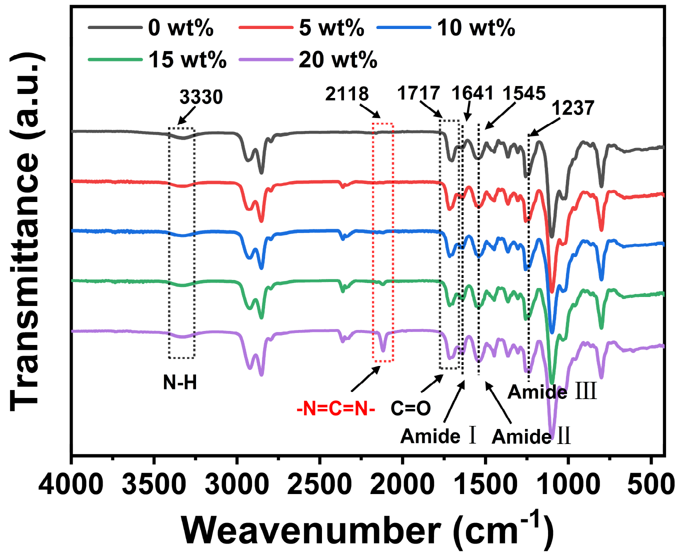

Figure S7. FTIR spectra of WE PUSX-C membranes with different PCC content.

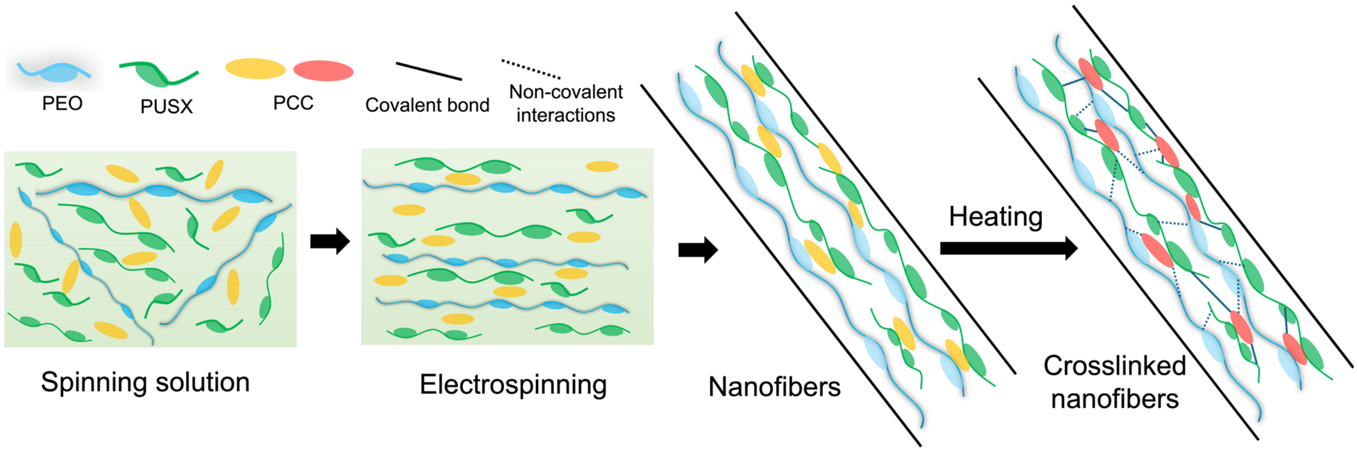

Figure S8. Schematic illustration of the formation of WE PUSX-C nanofibers via emulsion electrospinning. During the electrospinning process, under the action of a high-voltage electric field, the molecular chains of PEO and PUSX gradually transitioned from a disordered state to an oriented arrangement, forming non-crosslinked primary nanofibers together with the crosslinking agent. After heat treatment, the carboxyl groups in PUSX underwent covalent crosslinking reactions with the carbodiimide groups in PCC, constructing a chemically crosslinked network structure. In addition to chemical crosslinking, non-covalent interactions such as hydrogen bonding and electrostatic forces may also exist between the functional groups in PUSX, PEO, and PCC, further enhancing the resulting in a certain degree of physical crosslinking.

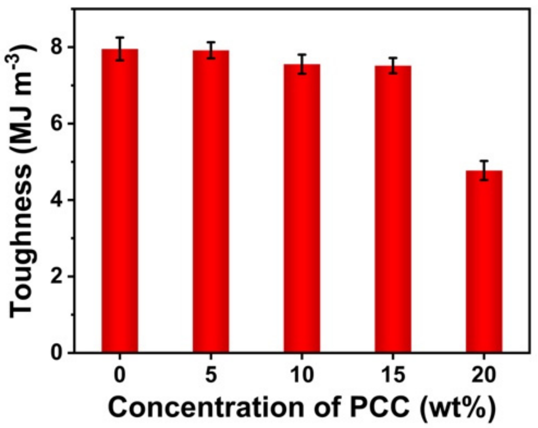

Figure S9. The toughness of WE PUSX-C membranes with different PCC content.

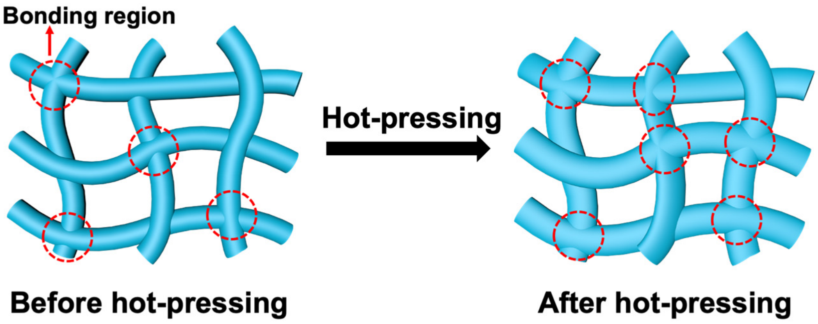

Figure S10. Hot-pressing mechanism diagram.

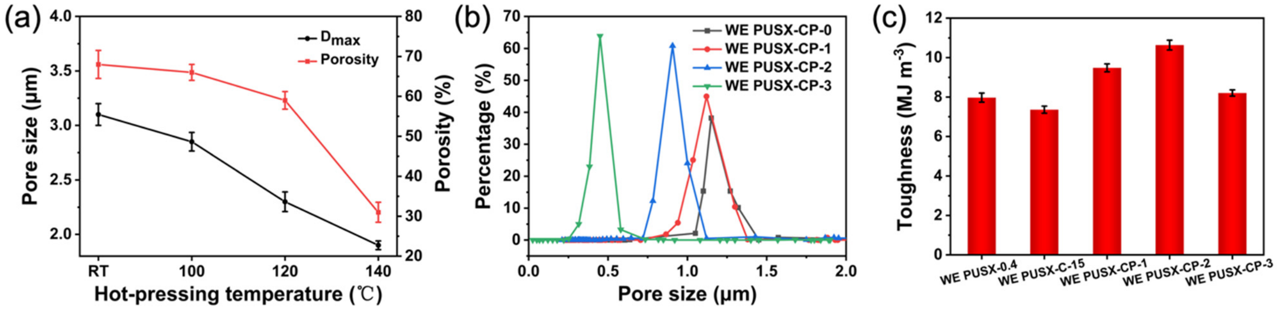

Figure S11. (**a**) pore size and porosity, (**b**) pore size distribution and (**c**) toughness of membrane with different hot-pressing temperature.

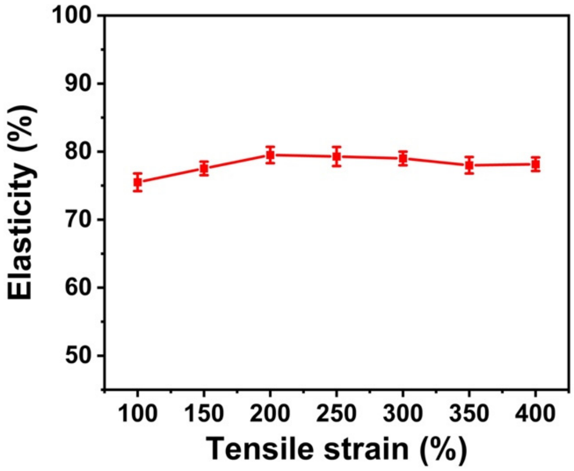

Figure S12. Elasticity of WE PUSX-CP-2 nanofibrous membranes under different tensile strains.

Table S1 A comparison of waterproof and breathable performances of WE PUSX-CP-2 membranes with other fluorine-free WBMs.

Fluorine-free WBMs	Material	WCA (°)	Hydrostatic pressure (kPa)	WVT rate (kg $m^{-2}d^{-1}$)	Tensile strength (MPa)	Toughness (M J m^{-3})	Elasticity (%)	Ref.
Water based	PUSX	137.9	51.5	5.48	3.85	10.37	~ 67.5 (400%, 160 cycles)	This work
	WPU/LAP	137.1	35.9	4.89	2.3	4.6	56.9 (300%, 100 cycles)	[1]
Organic solvent based	PU	----	3.7	9.0	----	----	----	[61]
	PA/PDMS	134.1	28.3	3.77	6.9	6.8	~ 46.0 (100%, 100 cycles)	[24]
	PU/HSG	142	5.45	8.05	6.3	----	----	[19]
	PU/PMHS	130.2	54.1	9.5	14.1	----	----	[16]
	PVB/PDMS	133.1	54.3	8.9	4.95	3.10	----	[62]
	SBS	128.3	63.9	4.54	3.72	----	----	[54]
